# Give Them a Word

**Published:** 1874-01

**Authors:** 


*Give them a Word.*

Will our editorial staff, one and all, as well as our friends generally do us the favor to present the claims of our advertising friends to the consideration of their home druggists and others?

